# Effects of nitrogen addition and plant litter manipulation on soil fungal and bacterial communities in a semiarid sandy land

**DOI:** 10.3389/fmicb.2023.1013570

**Published:** 2023-03-27

**Authors:** Jin Zhan, Yulin Li, Xueyong Zhao, Hongling Yang, Zhiying Ning, Rui Zhang

**Affiliations:** ^1^Naiman Desertification Research Station, Northwest Institute of Eco-Environment and Resources, Chinese Academy of Sciences, Lanzhou, China; ^2^College of Resources and Environment, University of Chinese Academy of Sciences, Beijing, China

**Keywords:** soil microbial community, plant, nitrogen addition, litter manipulation, Horqin sandy land

## Abstract

The plant and soil microbial communities are influenced by variability in environmental conditions (e.g., nitrogen addition); however, it is unclear how long-term nitrogen addition and litter manipulation affect soil microbial communities in a semiarid sandy grassland. Therefore, we simulated the impact of N addition and litter manipulation (litter removal, litter doubling) on plant and soil microbial communities in Horqin grassland, northern China through an experiment from 2014 to 2019. Our results revealed that in the case of non-nitrogen (N0), litter manipulation significantly reduced vegetation coverage (V) (*p* < 0.05); soil bacterial communities have higher alpha diversity than that of the fungi, and the beta diversity of soil fungi was higher than that of the bacteria; soil microbial alpha diversity was significantly decreased by nitrogen addition (N10) (*p* < 0.05); N addition and litter manipulation had significantly interactive influences on soil microbial beta diversity, and litter manipulation (C0 and C2) had significantly decreased soil microbial beta diversity (*p* < 0.05) in the case of nitrogen addition (N10) (*p* < 0.05). Moreover, bacteria were mostly dominated by the universal phyla Proteobacteria, Actinobacteria, and Acidobacteria, and fungi were only dominated by Ascomycota. Furthermore, the correlation analysis, redundancy analysis (RDA), and variation partitioning analysis indicated that the soil fungi community was more apt to be influenced by plant community diversity. Our results provide evidence that plant and soil microbial community respond differently to the treatments of the 6-year N addition and litter manipulation in a semiarid sandy land.

## 1. Introduction

Plant and soil microorganisms play important roles in regulating food and timber production, soil carbon (C) sequestration, and nutrient cycling in terrestrial ecosystems (Cui et al., 2020; Delgado-Baquerizo et al., 2020). The plant and soil microbial community is highly vulnerable to environmental changes in the context of global changes, such as increased atmospheric N deposition and land desertification (Liu et al., 2011, 2020; Zuo et al., 2012; Guo et al., 2019). Horqin sandy land is a typical semiarid sandy land in northern China, with relatively high dry N deposition (Lue and Tian, 2007). Due to the effect of strong wind in the process of land desertification, the spatial distribution pattern of litter on the ground surface takes place secondary distribution. Research showed that increased atmospheric N deposition and uneven litter distribution are likely to solely or interactively affect various biotic factors [e.g., plant aboveground biomass (AB), plant productivity, plant functional group composition, soil respiration (Zhao X. et al., 2020), microbial respiration] and abiotic factors (e.g., soil temperature, soil moisture, soil microbial biomass C, and soil microbial biomass N) (Shen et al., 2016; Liu et al., 2017; Gao et al., 2018; Gamadaerji et al., 2020; Zhao X. et al., 2020). Estimating the ecological consequences of N deposition and uneven litter distribution, as well as determining the influence on plant and soil microbial community in a semiarid sandy land are thus urgently needed.

N deposition has been an important component in the global N cycle, and anthropogenic N emission was increased since the industrial revolution (Galloway et al., 2008; Liu et al., 2011). China has become the third largest N deposition area since the late 1980s or early 1990s (Goulding et al., 1998). At the same time, N is the major limiting factor for plant growth in most terrestrial ecosystems (Gamadaerji et al., 2020), and a large number of studies have shown that increased atmospheric N deposition can increase plant community productivity, relieve nutrient limitation of microorganisms, promote activity of microorganisms, influence the bacterial and fungal community compositions, and accelerate the decomposition of litter, but significantly decrease plant species diversity in a community (Clark and Tilman, 2008; Isbell et al., 2013; Yue et al., 2016; Wang et al., 2017; Wang J. Q. et al., 2021). Some studies indicated that N deposition led to a change in plant functional group composition in a semiarid grassland (Gamadaerji et al., 2020).

Litter is one of the important carbon pools in the terrestrial ecosystem, and its decomposition process, as an important nutrient release pathway, connects the aboveground and underground carbon cycle process (Sayer et al., 2011). Global changes (e.g., N deposition), human activities, and changes in land use patterns are significantly affecting terrestrial ecosystem net primary production (NPP) and altering aboveground litter input to soil (LeBauer and Treseder, 2008; Fang et al., 2018). Previous studies showed that the litter quantity and decomposition rate would directly affect the process of nutrient absorption and utilization by plants and soil microorganisms, and further regulated the structure and function of an ecosystem (Wardle et al., 2004; Zhang et al., 2018). Focusing on sandy ecosystems, studies have shown that litter crusts can promote nutrient cycling in sandy ecosystems by mediating the restoration of bacterial taxa, rather than fungi, to enhance soil nutrient availability (Liu X. et al., 2021; Liu Y. et al., 2021). So the responses of soil microbial community to litter alteration in different terrestrial ecosystems has not yet reached a universal conclusion. One study indicated that N deposition affected litter decomposition by affecting soil N availability, litter yield and quality, soil biological factors, and factors in relation to litter decomposition (Yang et al., 2017). It’s well known that N addition promotes plant growth and increases litter production. It is noteworthy that when litter and N addition work collectively, how will it affect the plant and soil microbial community in the semiarid sandy land ecosystem?

Previous studies have shown that increased litter input and N addition significantly increased the community productivity in a semiarid grassland (Gamadaerji et al., 2020), litter addition suppressed the AB responses to N addition under ambient precipitation conditions by affecting soil moisture (Shen et al., 2016), and increased N deposition slightly weakened the inhibition of litter removal on soil respiration (Zhao X. et al., 2020). The biomass of perennial bunch grass (PB) and perennial rhizome grasses (PR) increased significantly with the increment of litter and nitrogen in a semiarid grassland after 6 year observation (Gamadaerji et al., 2020). Study on nitrogen and litter addition showed that litter addition increased AB and belowground net primary productivity (BNPP) in Inner Mongolia grassland after a two-year observation (Shen et al., 2016). In summary, when studying the impact of N addition and litter manipulation on plant and soil microbial communities, most researchers have focused on a single factor [e.g., N addition (Hou et al., 2021)] and have analyzed only one community [e.g., either bacteria or fungi, (van Diepen et al., 2017; Liu et al., 2020)]. But it is unknown how plant and microbial communities respond to N addition and litter manipulation in consecutive years. Thus, in this study, we examined the impact of N addition and litter manipulation (litter removal, litter doubling) on plant and soil microbial communities in a semiarid sandy land in northern China from 2014 to 2019. We hypothesized that (1) N addition can significantly affect plant and soil microorganisms, while litter treatment has interaction with N addition on the plant and soil microbial communities. (2) Fungal communities were more susceptible to N addition and litter treatment than that of bacteria in this semiarid sandy land over these 6 years. (3) The effect of plant community on soil fungal and bacterial community under N addition and litter manipulation was significantly different.

In this work, on the one hand, we can fill in the knowledge gap of the impact of long-term nitrogen addition and litter treatment on soil microbial communities in semi-arid sandy land. And on the other hand, we can improve our understanding of how sandy land ecosystems in semi-arid areas respond to environmental changes. It will provide reference for the follow-up research on the soil-plant-microbe interaction mechanism in semi-arid areas under long-term nitrogen deposition and aboveground litter change treatments.

## 2. Materials and methods

### 2.1. Study area

The study was conducted at a semiarid sandy land near Inner Mongolia Naiman Agroecosystem National Observation and Research Station (42°55′N, 120°41′E), in the southwestern part of the Horqin sandy land. The mean annual temperature is between 6.4 and 6.9°C. The mean annual precipitation is between 343 and 451 mm, with > 75% falling in the growing season (May to September). The mean annual wind speed is from 3.5 to 4.5 ms^–1^, the windy days are between 20 and 60 days, and sandstorms often occur in spring (about 10–15 days). It is mostly northwest wind in winter and spring and southwest in summer. The local geomorphic types are dominated by slowly-fluctuating sandy lands, with fixed dunes, semi-mobile dunes, mobile dunes, and inter-dune lowlands (Zhao et al., 2008). Affected by climate change and human activities, the region has experienced a different degree of desertification (Zuo et al., 2012; Zhao et al., 2014). The zonal soils are sandy chestnut soils with loose structures and vulnerable to wind erosion (Li Y. L. et al., 2016; Zuo et al., 2018). The soil is poor and mostly aeolian soil, and in the 0–30 cm soil layer, the soil nitrogen content was 0.057∼0.199 g.kg^–1^, the soil bulk density was 1.29∼1.59 g.cm^–3^ (Mao et al., 2012). In the 0–10 cm soil layer, the soil bulk density was about 1.41 g.cm^–3^, total nitrogen was about 0.57 g.kg^–1^, total carbon was about 4.16 g.kg^–1^ (Lv et al., 2021), and organic carbon was about 1.72 g.kg^–1^ (Chen et al., 2016). The dominant plant species are *Cleistogenes squarrosa* (Trin.) Keng, *Chenopodium acuminatum* Willd, *Pennisetum centrasiaticum* Tzvel, and *Setaria viridis* (L.) Beauv.

### 2.2. Experiment design

The field experiment of N addition and litter manipulation was established in 2014. The experiment was conducted in a randomized block design of six replicate blocks. In each block, we created six 10 m × 10 m plots and the plots were all spaced by 1 m apart to avoid cross effects between treatments. In this study, we only selected the experimental plots treated with nitrogen addition and litter manipulation for observation (Supplementary Figure 1). For the purpose of this study the experimental platform had set up 3 L manipulation levels [litter removal (C0), control (C1) and litter doubled (C2)] and 2 N addition levels [non-nitrogen addition (N0) and nitrogen addition (N10)], and composed of 6 treatments: N0C0, N0C1, N0C2, N10C0, N10C1, N10C2. So every treatment has six replicates. According to a typical practice of N treatment in many scientific simulation experiments of N deposition (Hasselquist et al., 2012; Gao et al., 2018), the experiment of N addition had been conducted at the beginning of May each year in the form of urea [CO(NH_2_)_2_] and the amount of N addition was 10 g N m^–2^ year^–1^. The urea was dissolved in 10 L purified water and sprayed evenly to each nitrogen addition (N10) plot, with a backpack sprayer. An equal amount of water was applied to the non-nitrogen addition (N0) areas. The experiment of litter manipulation was implemented at the end of November each year including N0 and N10. C0 has removed all litter materials on the soil surface, C1 was in a natural condition and remained intact. C2 has evenly added the litter collected in C0 to the corresponding plot of the same size. The main species of litter collected are *Artemisia scoparia* Waldst. et Kit., *P. centrasiaticum* Tzvel., *S. viridis* (L) Beauv., and *C. squarrosa* (Trin.) Keng.

### 2.3. Soil sampling

By the end of the plant growth season in September 2019, we collected 5 subsamples of soil at a depth of 10 cm using a soil corer with a diameter of 2.5 cm in each plot. The 5 samples were thoroughly mixed into a composite sample in the field, and then sieved with a 2 mm mesh to remove any roots or stones. The soil was stored at −80°C for analyzing the bacterial and fungal communities.

### 2.4. Measurement of plant community

At each block, 1 m × 1 m plots were established for vegetation surveys, and we surveyed plant species richness (S) and vegetation coverage (V) in September 2019. Four general diversity indexes are selected for calculation and analysis of plant diversity: Species richness (S, Equation 1), Shannon–Wiener’s diversity index (H, Equation 2), Pielou’s evenness index (E, Equation 3), and Simpson’s dominance index (λ, Equation 4) to evaluate plant community characteristics (Zhan et al., 2019). The calculation equations are:


(1) S=plant⁢species⁢in⁢the⁢sample⁢plot

(2) Shannon-Wiener′sdiversityindex(H):=-ΣPlnPii

(3) $${\mathrm{Pielou}}^{\prime }\,s\,\mathrm{evenness}\,\mathrm{index}\,\left(E\right)=H/\mathrm{lnS}$$

(4) $${\mathrm{Simpson}}^{\prime }\,s\,\mathrm{dominance}\,\mathrm{index}\,\left(\lambda \right)=1-\sum_{i=1}^{s}P_{i}^{2}$$

where, *P*_*i*_ is the ratio of the number of individuals of the i-th species in the 1 m × 1 m plots to the total number of all species in the sample plot (Zhan et al., 2019; Zhou et al., 2021).

### 2.5. Measurement of soil microbial community

Total genomic DNA was extracted from 0.4 g of well-mixed soil using the Power Soil DNA Isolation Kit (MoBio Laboratories, Carlsbad, USA) in accordance with the manufacturer’s specifications. Total genomic DNA was subjected to high-throughput sequencing using an IlluminaMiSeq platformat the Novogene Cooperation (Beijing, China). A detailed procedure can be found in the Supplementary information (Supplementary measurement of soil microbial community).

### 2.6. Statistical analysis

Statistical analyses were performed using SPSS22.0 (USA). We used multi-factor variance analysis to test the significance of the impact of N addition and litter manipulation on plant and soil microbial communities. Then, the effect of different litter manipulation levels on all response variables was tested using one-way ANOVA, and the effect of N addition levels on all response variables was tested by independent sample *t*-tests. Significant differences were assessed at *p* < 0.05. We performed non-metric multidimensional scaling (NMDS) analysis of soil microbial communities through the vegan package in R (version 3.6.2) using Bray–Curtis. Furthermore, we performed analysis of similarities (ANOSIM) to test whether the responses of microbial community profiles were significant through the vegan package in R (version 3.6.2). The correlation analysis of plant community diversity with soil microbial alpha diversity and community composition was conducted by OriginPro2021 (USA). The redundancy analysis (RDA) was performed using the “vegan” package in R (version 3.6.2), and *p*-values of the influence of each plant factor on soil microbial species distribution were calculated by the Envifit function. Variation partitioning analysis (VPA) was performed to determine the relative contributions of vegetation coverage, species richness, and plant diversity indexes (H, E and λ) to shaping the soil fungal and bacterial community composition.

## 3. Results

### 3.1. Responses of plant community diversity to N addition and litter manipulation

N addition and litter manipulation had no significantly interactive influences on plant community diversity indexes at the end of 6-year (Supplementary Figure 2). Compared with non-nitrogen (N0), the nitrogen addition (N10), respectively, increased vegetation coverage (V), Pielou’s evenness (E) and Simpson’s dominance (λ) index by 7.16, 16.84, and 5.36% (Supplementary Figures 2A, C, E), and decreased species richness (S) and Shannon–Wiener’s diversity (H) by 15.06 and 2.46% (Supplementary Figures 2B, D). Compared with litter control (C1), litter removal (C0) greatly reduced vegetation coverage (V) and species richness (S) but increased Pielou’s evenness (E) index (Supplementary Figures 2A–C). Furthermore, in the case of non-nitrogen (N0), litter manipulation significantly reduced vegetation coverage (V) (Supplementary Figure 2A, *p* < 0.05).

### 3.2. Responses of soil microbial alpha and beta diversity to N addition and litter manipulation

**FIGURE 1:**
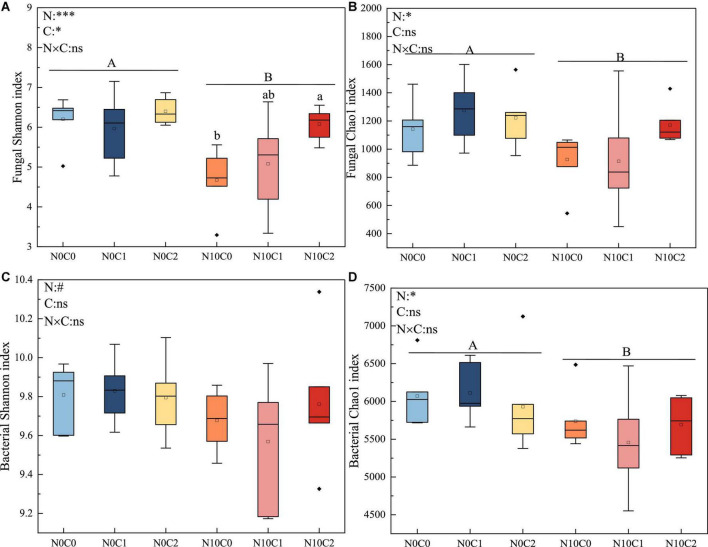
The 6-year of soil fungal and bacterial Shannon diversity index and chao1 richness in N addition and litter manipulation treatments during 2014–2019. Significance levels were presented to show the effect of N addition (N) and altered litter input (C) treatments and their interaction (N × C) on these parameters (^ns^*p* > 0.1; ^#^*p* < 0.1; *p < 0.05; **p < 0.01; ***p < 0.001). The uppercase letters indicate the significant difference between two nitrogen treatments (*p* < 0.05), different lowercase letters indicate significant difference among three altered litter input treatments in the case of same nitrogen (*p* < 0.05). **(A–D)** Represents the α diversity indices of soil fungi and bacteria (Shannon index and Chao1 index), respectively.

Microbial alpha diversity was used to describe the composition of microbial community for a single habitat or treatment, and beta diversity was used to describe the assembly of microbial communities among different habitats or treatments. In our study, the alpha diversity of fungi was lower than that of bacteria. N addition and litter manipulation had no significantly interactive influences on soil fungal and bacterial alpha diversity at the end of 6-year (Figure 1 and Supplementary Figure 3). Nitrogen addition (N10) significantly reduced fungal alpha diversity (Figure 1 and Supplementary Figure 3, *p* < 0.05). Similarly, nitrogen addition (N10) significantly decreased bacterial Simpson, chao1, and Coverage estimators based on abundance (ACE) index (Figure 1 and Supplementary Figure 3, *p* < 0.05). Furthermore, in the case of nitrogen addition (N10), Shannon index was increased by litter doubling (C2), but decreased by litter removal (C0) in fungi (Figure 1A, *p* < 0.05).

**FIGURE 2:**
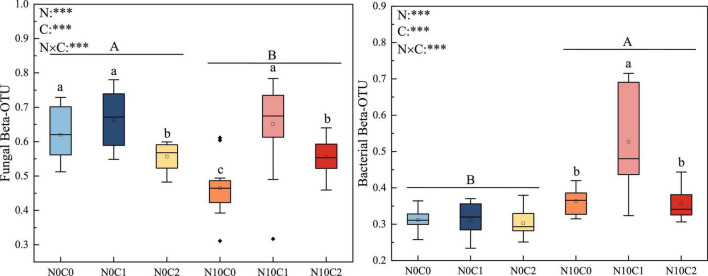
The 6-year of soil fungal and bacterial beta diversity in N addition and litter manipulation treatments during 2014–2019. Significance levels were presented to show the effect of N addition (N) and altered litter input (C) treatments and their interaction (N × C) on these parameters (^ns^*p* > 0.1; ^#^*p* < 0.1; *p < 0.05; **p < 0.01; ***p < 0.001). The uppercase letters indicate the significant difference between two nitrogen treatments (*p* < 0.05), different lowercase letters indicate significant difference among three altered litter input treatments in the case of same nitrogen (*p* < 0.05).

**FIGURE 3:**
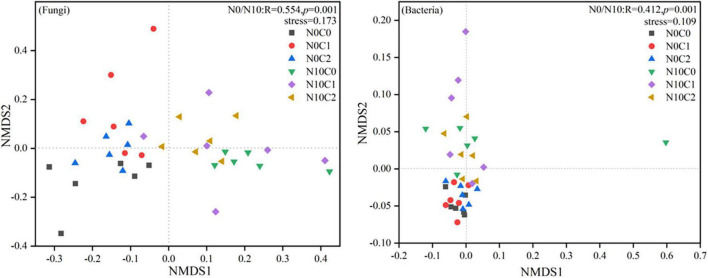
Non-metric multidimensional scaling (NMDS) analysis of soil fungal and bacterial beta diversity in N addition and litter manipulation treatments during 2014–2019. R and *p*-values under the non-nitrogen (N0) and nitrogen addition (N10) treatments comes from the ANOSIM analysis.

The beta diversity of soil fungi was higher than that of bacteria. In fungi, beta diversity was significantly reduced by nitrogen addition (N10) (Figure 2, *p* < 0.05). In contrary, nitrogen addition (N10) significantly increased soil bacteria beta diversity (Figure 2, *p* < 0.05). The beta diversity of fungi and bacteria were significantly decreased by litter manipulation (C0 and C2) (Figure 2, *p* < 0.05). N addition and litter manipulation had significantly interactive influences on soil fungal and bacterial beta diversity at the end of 6-year (Figure 2, *p* < 0.05). In the case of non-nitrogen addition (N0), the beta diversity of fungi was significantly decreased by litter doubling (C2) (Figure 2, *p* < 0.05). Furthermore, in the case of nitrogen addition (N10), the beta diversity of fungi and bacteria were significantly decreased by litter manipulation (C0 and C2) (Figure 2, *p* < 0.05). To further explore the differences in beta diversity, NMDS analysis was carried out. In fungi and bacteria, the Bray–Curtis dissimilarity from the nitrogen addition (N10) and non-nitrogen (N0) treatments was separated along the *X*- or *Y*-axis (Figure 3, stress = 0.173, stress = 0.109). Furthermore, the ANOSIM highlighted that the soil fungal and bacterial communities with the nitrogen addition (N10) were substantially different from those of non-nitrogen (N0) treatments (*p* < 0.05). The clear differences in soil fungal and bacterial communities under N addition and litter manipulation during 2014–2019 were shown in Supplementary Table 1.

### 3.3. Responses of soil microbial community composition to N addition and litter manipulation

**FIGURE 4:**
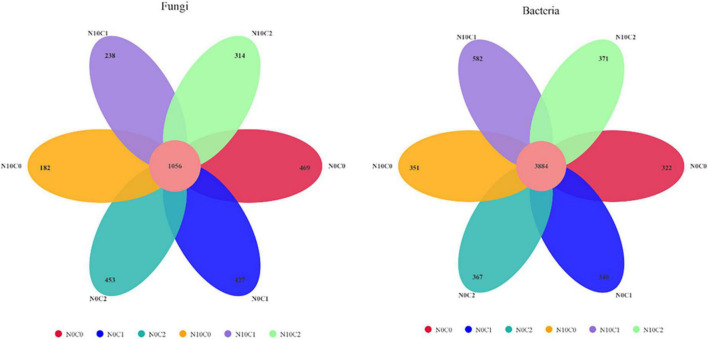
Venn diagram of the soil fungal **(left)** and bacterial **(right)** OTU in N addition and litter manipulation treatments during 2014–2019. The numbers within circles represent the specific OTU in that treatment, the core number represents the common OTU present in all treatment.

The Venn diagrams represent the numbers of specific bacterial and fungal species (represented by OTUs) associated with different treatments (Figure 4). A total of 3,139 soil fungal operational taxonomic unit (OTU) species and 6,217 bacteria were detected in our study. In the case of non-nitrogen addition (N0), a greater number of treatment-specific fungal species were detected in each treatment than bacterial species (Figure 4). In fungi, the core common species dominated the study area and represented 69.25∼85.30%. In addition, compared with non-nitrogen (N0), nitrogen addition (N10) reduced the OTU species from 1,505.67 to 1,300.67 (Figure 4 fungi). In comparison, the core common bacterial species accounted for 86.97∼92.34%, whereas there was little difference between nitrogen addition (N10) and non-nitrogen (N0) (Figure 4 bacteria).

**FIGURE 5:**
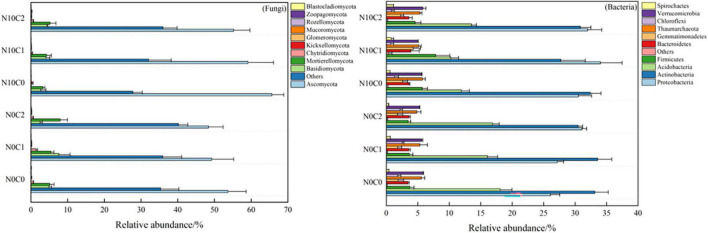
Soil microbial community proportion at phylum level in N addition and litter manipulation treatments during 2014–2019 (mean).

N addition and litter manipulation had an influence on the relative abundance of many common taxa (top 10%) at the phylum level in soil fungi and bacteria (Supplementary Table 2). Soil fungal communities changed lesser along with different treatments than bacteria. Specifically, the Ascomycota (49.20∼65.63%) was relatively dominant in fungi (Figure 5 fungi), then the Basidiomycota was 2.50∼7.61% and Mortierellomycota was 3.29∼7.96%. In comparison, the bacterial community was getting more diverse among different treatments. The Proteobacteria (26.08∼34.03%), Actinobacteria (27.73∼33.58%), Acidobacteria (10.24∼18.09%), and Firmicutes (3.46∼7.83%) were the most abundant bacterial phyla (Figure 5 bacteria). Meanwhile, the result of the independent sample *t*-test for N addition treatment showed that nitrogen addition (N10) treatment significantly increased the relative abundance of Ascomycota, but decreased the relative abundance of Chytridiomycota (*p* < 0.05) in fungi (*p* < 0.05) (Supplementary Figures 4A, B, *p* < 0.05). Similarly, nitrogen addition (N10) treatment significantly increased the relative abundance of Proteobacteria, Firmicutes, Bacteroidetes, and Thaumarchaeota (Supplementary Figures 4C, D, *p* < 0.05), but decreased the relative abundance of Acidobacteria (*p* < 0.05) in bacteria (Supplementary Figure 5, *p* < 0.05).

### 3.4. Relationship between the soil microbial community and plant community diversity under N addition and litter manipulation

The correlation analysis result revealed that Shannon, chao1 and ACE indexes of fungi were positively correlated with species richness (S) (Supplementary Figure 6, *p* < = 0.05). Similarly, Simpson index of bacteria was positively correlated with species richness (S) (Supplementary Figure 6, *p* < = 0.05). Furthermore, the Pielou’s evenness (E) decreased significantly with an increasing relative abundance of Mucoromycota in fungi (Supplementary Figure 6 fungi, *p* < 0.05). Whereas vegetation coverage (V) increased significantly with the increasing relative abundance of Gemmatimonadetes in soil bacteria (Supplementary Figure 6 bacteria, *p* < 0.05).

**FIGURE 6:**
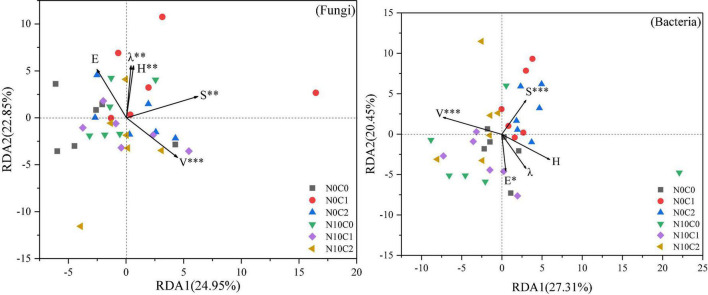
Redundancy analysis (RDA) of plant community diversity and soil microbial species distribution in community under N addition and litter manipulation. Significance levels of each plant community factor were calculated by Envifit function (^ns^*p* > 0.1; ^#^*p* < 0.1; *p < 0.05; **p < 0.01; ***p < 0.001).

The effect of N addition and litter manipulation on soil microbial community was analyzed using RDA (Figure 6). The cumulative percentage variance of the first and second axes was 24.95 and 22.85% for fungi, and 27.31 and 20.45% for bacteria, respectively, indicating that the soil microbial species distribution was significantly affected by the plant community diversity (Figure 6). And the Envifit function results indicated that species richness (S) and vegetation coverage (V) were the most important influential factors to the changes in soil microbial species distribution (*p* < 0.01). Furthermore, Shannon–Wiener’s diversity (H) and Simpson’s dominance (λ) had significant effect on soil fungal species distribution (*p* < 0.01), and Pielou’s evenness (E) had a significant effect on soil bacterial species distribution (*p* < 0.05).

**FIGURE 7:**
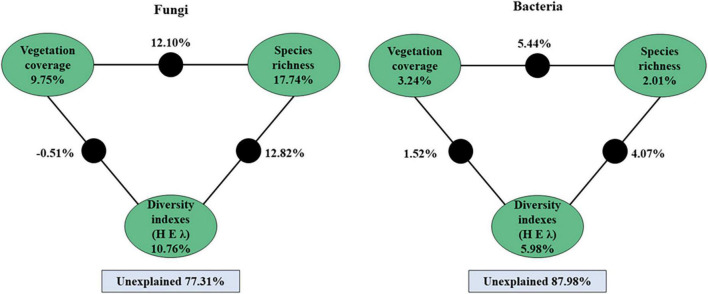
Variation partitioning analysis (VPA) of the fungal and bacterial community explained by vegetation coverage, species richness and plant’s diversity indexes (H, E, and λ) and their interactions. The value of black circle is the common explanation of the factors at both ends.

A variation partitioning analysis further demonstrated that the soil bacterial and fungal communities were highly explained by vegetation coverage, species richness, and diversity indexes (Figure 7). For fungi, a total of 22.69% of the community variation could be explained by plant variables, and the species richness explained 17.74% of the variation. For bacteria, a total of 12.02% of the community variation could be explained by plant variables, and plant diversity indexes explained 5.98% of the variation (Figure 7).

## 4. Discussion

### 4.1. Effects of N addition and litter manipulation on plant community diversity

In our study, nitrogen addition (N10) had no significant influences on vegetation coverage (V) and plant community diversity, and there are small effects. The reason, on the one hand, N is a key limiting factor in the growth of plants in arid and semiarid regions, and the required nitrogen for plant growth mainly comes from the soil or plant’s nitrogen fixation (Yang et al., 2017). Nitrogen addition will ease the limiting factor, increase the absorption of CO_2_ in the atmosphere by plants, significantly improve photosynthesis efficiency (Gao Y. et al., 2013), and is conducive to plant growth. So vegetation coverage (V), Pielou’s evenness (E) index, and Simpson’s dominance (λ) were increased in the semiarid sandy land. On the other hand, the addition of nitrogen greatly intensifies interspecific competition in the plant community, tall plants produce photoinhibition to short plants (Li C. B. et al., 2016), at the same time, the increase of plant vegetation coverage (V) under nitrogen addition weakens the surface solar radiation (Kan et al., 2015; Zhao X. X. et al., 2020), which is not conducive to the growth of bottom plants. So nitrogen addition (N10) reduced species richness (S) and Shannon–Wiener’s diversity (H). The plant community did not show significant difference at the end of the 6-year N addition in the semiarid sandy land. The discrepancies could due to that plant diversity responses may also vary depending on the N deposition levels (Lu et al., 2011), different plants with divergent N utilization (Gherardi et al., 2013) and experimental duration (De Schrijver et al., 2011).

Our study presented that litter removal (C0) greatly reduced vegetation coverage (V) and species richness (S). In contrast, litter doubling (C2) slightly increased vegetation coverage (V) and species richness (S) in nitrogen addition (N10), and slightly reduced vegetation coverage (V) and species richness (S) in non-nitrogen (N0). The results addressed part of our first hypothesis: litter manipulation had no significant influence on the plant community. The explanation for the observed differences might be that: First, litter removal (C0) inhibited seed activity of some species or prolonged seed dormancy by increasing soil temperature and reducing soil moisture, thereby significantly inhibiting seed germination (Cuena-Lombrana et al., 2016). Second, litter removal (C0) increased near-surface photosynthetically active radiation, accelerated water loss, reduced photosynthetic rate, and indirectly increased seedling mortality (Bajwa et al., 2017; Zhang, 2019). So litter removal (C0) greatly reduced vegetation coverage (V) and species richness (S). Third, nitrogen addition alleviated the N limitation of plant growth in this area, and litter doubling (C2) slowed evaporation of soil surface moister, promoted the growth of annual species in semiarid areas, and hence increased vegetation coverage (V) and species richness (S). However, in the non- nitrogen addition, the smaller seeds of one or two annual plants stayed in the litter layer (Wang et al., 2013), leading to delay or failure of germination and reducing vegetation coverage (V) and species richness (S).

### 4.2. Effects of N addition and litter manipulation on soil microbial diversity

Bacteria has higher alpha diversity than fungi in many ecosystems (Anderson et al., 2017; Chen et al., 2020). Our study confirmed this statement in the semiarid sandy land, and fungal alpha and beta diversity were significantly decreased by nitrogen addition (*p* < 0.05). In fact, fungi typically has lower nutrient requirements than bacteria (Zhou et al., 2017). By contrast, bacteria grow faster and prefer substrates of low C: N ratios. Plants provide soil microbes with C in exchange for other soil nutrients, such as N (Vasar et al., 2017). N availability is increased due to N deposition in soil, plants release lesser amounts of C to soil microbes (Johnson and Thornley, 1987), lesser C could be allocated to belowground parts, resulting in a decrease in the soil C storage in a long term. Consequently, with the addition of nitrogen, when the soil C/N ratio was low, the effect on fungi is greater than bacteria. Our study showed that soil bacterial and fungal alpha and beta diversity index changed to different degrees under nitrogen addition. A explanation for the discrepancies may be the fact that bacteria and fungi are the two major microbial taxa in soils, and they respond differently concerning their morphological traits, utilization strategies, and sensitivities to the environment (Chen et al., 2020).

Litter accumulation can promote the formation of soil organic carbon and affect the community of soil microorganisms (Wang L. Y. et al., 2021). In our study, litter manipulation (C) had no significant influence on soil microbial alpha diversity. This is inconsistent with some studies showing that after the removal or addition of litter in soil, the alpha diversity indexes of soil microbial community have changed significantly (Wang L. Y. et al., 2021). One study indicated that the input of plant litter did not affect the soil organic carbon content (SOC) content in the observation of two temperate forests, even after 11 years of treatment (Holub et al., 2005). And one study indicated that the underground root system may provide a more stable carbon source for the soil than the above-ground litter (Sokol et al., 2019). Therefore, we should consider the influence of underground litter and/or the coupled impacts with the above ground litters on soil microbial community in the later study.

### 4.3. Effects of N addition and litter manipulation on soil microbial community composition

The microbial OTU taxonomic composition excavates the specific difference along with environmental change. As our Venn diagrams indicated that the core common species accounted for 86.97∼92.34% of the total OTU species in soil bacteria, which were less specific and more adaptable along with environmental change than the fungal species that the core common species represented 69.25∼85.30% of the total soil fungi. In addition, Ascomycota’s proportion was greater than 48% in fungal at the phylum level. Proteobacteria, Actinobacteria, and Acidobacteria were relatively dominant in bacteria. These results confirmed that most soil bacterial taxa occupied a wide range, On the other hand, most soil fungal taxa occupied a narrower range. Therefore, soil bacteria adapted to the environment by changing the proportions of their taxa, while fungi changed their rare taxa in response to environmental change. This was consistent with the results that soil bacterial and fungal communities adapted to the natural aridity gradient in desert grassland ecosystems (Wang S. K. et al., 2021).

Furthermore, nitrogen addition (N10) significantly increased the relative abundance of Ascomycota, Proteobacteria, Firmicutes, Bacteroidetes, and Thaumarchaeota (*p* < 0.05), but significantly decreased the relative abundance of Acidobacteria (*p* < 0.05). There are several possible explanations for this result. On the one hand, nutrient enrichment may alter the interactions among microbial species, shifting from symbiosis to competition with increased nutrient supply (Hoek et al., 2016), leading to changes in soil microbial community composition. On the other hand, as the fact that, microbial taxa can have niche preferences, even within the same phylum (Faust and Raes, 2012). For example, our study showed that in semi-arid sandy land, continuous 6 years nitrogen addition was beneficial to Copiotrophic taxa, Proteobacteria’s reproduction, but was not apt to oligotrophic taxa Acidobacteria’s survival.

### 4.4. Relationship of plant community diversity with soil microbial community under N addition and litter manipulation

Plant species are the main driving factor for microenvironmental change, affecting soil nutrient availability (Schiedung et al., 2017) and ultimately the microbial community (Zuo et al., 2016; Wang et al., 2018). We found that only the species richness (S) was significantly positively associated with some alpha diversity indices of soil microorganisms, and RDA showed that the soil microbial species distribution was significantly affected by the plant community diversity. A variation partitioning analysis further demonstrated that plant variables explained the change in soil fungal and bacterial community by 22.69 and 12.02%. These results indicated that plant community diversity influenced soil fungi more than bacteria. One explanation for this association is that shared environmental factors contribute to relationships between soil microorganisms and plant community composition (Prober et al., 2015). First, plants provide microhabitats as well as organic substrates for soil microorganisms, and such changes in plant community composition lead to changes in both habitats and carbon resources for soil microhabitats (Ramirez et al., 2012), and translating into changes in soil microbial communities. Second, changes in the soil microbial community can affect the plant community. For instance, plant-soil feedback is associated with the processes of soil organic matter decomposition and mineralization, or pathogenic and beneficial interactions (De Deyn and Van der Putten, 2005; Brigham et al., 2022). Third, fungi are often more directly dependent on plant products and mycorrhizal fungi are more dependent on direct symbiotic relationships with plants (Gao C. et al., 2013; Prober et al., 2015). As in this study, soil fungi Shannon, chao1 and ACE indices were significantly positively correlated with species richness (S).

Our results showed that N addition and litter manipulation had no significantly interactive influences on plant and soil microbial community except that soil microbial beta diversity. There are several possible explanations for this result. On the one hand, N addition was dependent on the water condition (Zong et al., 2019). The interannual variability of precipitation in the semi-arid area fluctuates greatly, which will affect the nitrogen use efficiency. On the other hand, as a nutrient provider, litter needs to accumulate in a certain time scale during its decomposition process and its nutrient utilization. Thence to further explore the interaction between N addition and litter manipulation, we need more long-term data accumulation or consideration of factors such as moisture, precipitation, and time in the data analysis process. In this work, we focused on sandy ecosystems in semi-arid regions and filled in the gaps in knowledge about the effects of nitrogen deposition and aboveground litter treatment on soil microbial communities on long-term scales. However, soil microbial communities are mainly regulated by multiple environmental factors, such as climate, topography, soil properties, and vegetation type (Bodelier, 2011; Yang et al., 2018). Nitrogen addition causes soil acidification, if at the end of nitrogen addition, accompanied by a large amount of natural precipitation, the semiarid sandy soil has poor water and fertilizer retention capacity and is prone to leaching, which will affect the utilization of nitrogen by plants and shallow soil microorganisms. So to more rigorously distinguish the effects of abiotic environments on plant communities and soil microbial communities, and the relationship between plant communities and soil microorganisms, it is necessary to conduct experiments that simultaneously manipulate plant communities, soil microorganisms, and related abiotic factors.

## 5. Conclusion

This study demonstrated that continued N addition and litter manipulation for 6 years had no significant interactive influences on plant and soil microbial community except for soil microbial beta diversity in a semiarid sandy land. Whereas soil microbial alpha and beta diversities were significantly decreased by nitrogen addition (*p* < 0.05) except for bacterial beta diversity. Furthermore, soil bacterial and fungal communities responded differently, bacterial communities showing higher alpha diversity than fungi, and the beta diversity of soil fungi higher than bacteria. In particular, soil bacteria were dominated by the universal phyla, while fungi were dominated only by the phylum Ascomycota. Results from our study also indicated that most soil bacteria (of the same taxa) occupied a wide range and adapted to the environment by changing the proportions of their taxa, while fungi changed their rare taxa in response to environmental change. Finally, the soil microbial species distribution was significantly affected by the plant community diversity. A variation partitioning analysis further demonstrated that plant variables explained the changes in soil fungal and bacterial communities by 22.69 and 12.02%.

## Data availability statement

The datasets presented in this study are deposited in the NCBI repository, accession number: PRJNA944160.

## Author contributions

YL, XZ, JZ, HY, and ZN conceived and designed the study. XZ, JZ, HY, and RZ performed the experiments. JZ analyzed the results and drafted the manuscript. All authors had a chance to review the manuscript before submission, contributed to the discussion and interpretation of the data, and approved the submitted version.

## References

[B1] Anderson C. Beare M. Buckley H. L. Lear G. (2017). Bacterial and fungal communities respond differently to varying tillage depth in agricultural soils. *Peerj* 5:e3930. 10.7717/peerj.3930 29062604 PMC5649590

[B2] Bajwa A. A. Akhter M. J. Iqbal N. Peerzada A. M. Hanif Z. Manalil S. (2017). Biology and management of *Avena fatua* and *Avena ludoviciana*: two noxious weed species of agro-ecosystems. *Environ. Sci. Pollut. Res.* 24 19465–19479. 10.1007/s11356-017-9810-y 28766148

[B3] Bodelier P. L. E. (2011). Toward understanding, managing, and protecting microbial ecosystems. *Front. Microbiol.* 2:80. 10.3389/fmicb.2011.00080 21747797 PMC3128941

[B4] Brigham L. M. Mesquita C. P. B. D. Smith J. G. Sartwell S. A. Schmidt S. K. Suding K. N. (2022). Do plant–soil interactions influence how the microbial community responds to environmental change? *Ecology* 103:e03554. 10.1002/ecy.3554 34622953

[B5] Chen J. Li Y. Feng J. Su N. Zhao X. (2016). Links of temperature and moisture with soil nitrogen mineralization in the horqin sandy grassland. *J. Desert Res.* 36 103–110.

[B6] Chen J. Wang P. F. Wang C. Wang X. Miao L. Z. Liu S. (2020). Fungal community demonstrates stronger dispersal limitation and less network connectivity than bacterial community in sediments along a large river. *Environ. Microbiol.* 22 832–849. 10.1111/1462-2920.14795 31469494

[B7] Clark C. M. Tilman D. (2008). Loss of plant species after chronic low-level nitrogen deposition to prairie grasslands. *Nature* 451 712–715. 10.1038/nature06503 18256670

[B8] Cuena-Lombrana A. Porceddu M. Dettori C. A. Bacchetta G. (2016). *Gentiana lutea* L. subsp lutea seed germination: natural versus controlled conditions. *Botany* 94 653–659. 10.1139/cjb-2016-0030

[B9] Cui H. Y. Sun W. Delgado-Baquerizo M. Song W. Z. Ma J. Y. Wang K. Y. (2020). The effects of mowing and multi-level N fertilization on soil bacterial and fungal communities in a semiarid grassland are year-dependent. *Soil Biol. Biochem.* 151 144–155. 10.1016/j.soilbio.2020.108040

[B10] De Deyn G. B. Van der Putten W. H. (2005). Linking aboveground and belowground diversity. *Trends Ecol. Evol.* 20 625–633. 10.1016/j.tree.2005.08.009 16701446

[B11] De Schrijver A. De Frenne P. Ampoorter E. Van Nevel L. Demey A. Wuyts K. (2011). Cumulative nitrogen input drives species loss in terrestrial ecosystems. *Global Ecol. Biogeogr.* 20 803–816. 10.1111/j.1466-8238.2011.00652.x

[B12] Delgado-Baquerizo M. Reich P. B. Trivedi C. Eldridge D. J. Abades S. Alfaro F. D. (2020). Multiple elements of soil biodiversity drive ecosystem functions across biomes. *Nat. Ecol. Evol.* 4 210–220. 10.1038/s41559-019-1084-y 32015427

[B13] Fang J. Yu G. Liu L. Hu S. Chapin F. (2018). Climate change, human impacts, and carbon sequestration in China. *Proc. Natl. Acad. Sci. U S A.* 115 4015–4020. 10.1073/pnas.1700304115 29666313 PMC5910806

[B14] Faust K. Raes J. (2012). Microbial interactions: from networks to models. *Nat. Rev. Microbiol.* 10 538–550. 10.1038/nrmicro2832 22796884

[B15] Galloway J. N. Townsend A. R. Erisman J. W. Bekunda M. Cai Z. Freney J. R. (2008). Transformation of the nitrogen cycle: recent trends, questions, and potential solutions. *Science* 320 889–892. 10.1126/science.1136674 18487183

[B16] Gamadaerji Z. Tan X. R. Wang S. S. Li W. J. You C. H. (2020). Effect of altered litter input and nitrogen addition on ecosystem aboveground primary productivity and plant functional group composition in a semiarid grassland. *Chin. J. Plant Ecol.* 44 791–806. 10.17521/cjpe.2020.0126

[B17] Gao C. Shi N. N. Liu Y. X. Peay K. G. Zheng Y. Ding Q. (2013). Host plant genus-level diversity is the best predictor of ectomycorrhizal fungal diversity in a Chinese subtropical forest. *Mol. Ecol.* 22 3403–3414. 10.1111/mec.12297 24624421

[B18] Gao Y. Yu G. R. He N. P. (2013). Equilibration of the terrestrial water, nitrogen, and carbon cycles: advocating a health threshold for carbon storage. *Ecol. Eng.* 57 366–374. 10.1016/j.ecoleng.2013.04.011

[B19] Gao Q. Bai E. Wang J. S. Zheng Z. M. Xia J. Y. You W. H. (2018). Effects of litter manipulation on soil respiration under short-term nitrogen addition in a subtropical evergreen forest. *For. Ecol. Manag.* 429 77–83. 10.1016/j.foreco.2018.06.037

[B20] Gherardi L. A. Sala O. E. Yahdjian L. (2013). Preference for different inorganic nitrogen forms among plant functional types and species of the Patagonian steppe. *Oecologia* 173 1075–1081. 10.1007/s00442-013-2687-7 23812108

[B21] Goulding K. W. T. Bailey N. J. Bradbury N. J. Hargreaves P. Howe M. Murphy D. V. (1998). Nitrogen deposition and its contribution to nitrogen cycling and associated soil processes. *New Phytol.* 139 49–58. 10.1046/j.1469-8137.1998.00182.x

[B22] Guo Q. X. Yan L. J. Korpelainen H. Niinemets Ü Li C. Y. (2019). Plant-plant interactions and N fertilization shape soil bacterial and fungal communities. *Soil Biol. Biochem.* 128 127–138. 10.1016/j.soilbio.2018.10.018

[B23] Hasselquist N. J. Metcalfe D. B. Hogberg P. (2012). Contrasting effects of low and high nitrogen additions on soil CO2 flux components and ectomycorrhizal fungal sporocarp production in a boreal forest. *Glob. Change Biol.* 18 3596–3605. 10.1111/gcb.12001

[B24] Hoek T. A. Axelrod K. Biancalani T. Yurtsev E. A. Liu J. H. Gore J. (2016). Resource availability modulates the cooperative and competitive nature of a microbial cross-feeding mutualism. *PLoS Biol.* 14:e1002540. 10.1371/journal.pbio.1002540 27557335 PMC4996419

[B25] Holub S. M. Lajtha K. Spears J. D. H. Toth J. A. Crow S. E. Caldwell B. A. (2005). Organic matter manipulations have little effect on gross and net nitrogen transformations in two temperate forest mineral soils in the USA and central Europe. *For. Ecol. Manag.* 214 320–330. 10.1016/j.foreco.2005.04.016

[B26] Hou S. L. Hattenschwiler S. Yang J. J. Sistla S. Wei H. W. Zhang Z. W. (2021). Increasing rates of long-term nitrogen deposition consistently increased litter decomposition in a semi-arid grassland. *New Phytol.* 229 296–307. 10.1111/nph.16854 32762047

[B27] Isbell F. Reich P. B. Tilman D. Hobbie S. E. Polasky S. Binder S. (2013). Nutrient enrichment, biodiversity loss, and consequent declines in ecosystem productivity. *Proc. Natl. Acad. Sci. U S A.* 110 11911–11916. 10.1073/pnas.1310880110 23818582 PMC3718098

[B28] Johnson I. R. Thornley J. H. M. (1987). A model of shoot-root partitioning with optimal growth. *Ann. Bot.* 60 133–142. 10.1093/oxfordjournals.aob.a087429

[B29] Kan Y. C. Wu R. X. Zhong M. Y. Wang J. X. Pu X. P. Shao X. Q. (2015). The response of net soil respiration to different disturbances in a typical grassland of northern China. *Acta Ecologica Sinica* 35 6041–6050. 10.5846/stxb201401060031

[B30] LeBauer D. S. Treseder K. K. (2008). Nitrogen limitation of net primary productivity in terrestrial ecosystems is globally distributed. *Ecology* 89 371–379. 10.1890/06-2057.1 18409427

[B31] Li C. B. Peng Y. F. Zhao D. Z. Ning Y. Zhou G. Y. (2016). Effects of precipitation change and nitrogen addition on community structure and plant diversity in an alpine steppe on the Qinghai-Tibetan plateau. *Res. Soil Water Conserv.* 23 185–191.

[B32] Li Y. L. Ning Z. Y. Cui D. Mao W. Bi J. D. Zhao X. Y. (2016). Litter decomposition in a semiarid dune grassland: neutral effect of water supply and inhibitory effect of nitrogen addition. *PLoS One* 11:e0162663. 10.1371/journal.pone.0162663 27617439 PMC5019385

[B33] Liu J. S. Zhang X. Wang H. Hui X. L. Wang Z. H. Qiu W. H. (2017). Long-term nitrogen fertilization impacts soil fungal and bacterial community structures in a dryland soil of Loess Plateau in China. *J. Soils Sediments* 18 1632–1640. 10.1007/s11368-017-1862-6

[B34] Liu W. X. Jiang L. Yang S. Wang Z. Tian R. Peng Z. Y. (2020). Critical transition of soil bacterial diversity and composition triggered by nitrogen enrichment. *Ecology* 101:e03053. 10.1002/ecy.3053 32242918

[B35] Liu X. Liu Y. Zhang L. Yin R. Wu G.-L. (2021). Bacterial contributions of bio-crusts and litter crusts to nutrient cycling in the Mu Us Sandy Land. *Catena* 199:105090. 10.1016/j.catena.2020.105090

[B36] Liu Y. Havrilla C. A. Jia C. Liu X.-Z. Wu G.-L. (2021). Litter crusts enhance soil nutrients through bacteria rather than fungi in sandy ecosystems. *Catena* 204:105413. 10.1016/j.catena.2021.105413

[B37] Liu X. J. Duan L. Mo J. M. Du E. Z. Shen J. L. Lu X. K. (2011). Nitrogen deposition and its ecological impact in China: an overview. *Environ. Pollut.* 159 2251–2264. 10.1016/j.envpol.2010.08.002 20828899

[B38] Lu X. Mo J. Gilliam F. S. Yu G. Zhang W. Fang Y. (2011). Effects of experimental nitrogen additions on plant diversity in tropical forests of contrasting disturbance regimes in southern China. *Environ. Pollut.* 159 2228–2235. 10.1016/j.envpol.2010.10.037 21122959

[B39] Lue C. Q. Tian H. Q. (2007). Spatial and temporal patterns of nitrogen deposition in China: synthesis of observational data. *J. Geophys. Res. Atmos.* 112:D22S05. 10.1029/2006JD007990

[B40] Lv P. Sun S. Medina-Roldand E. Zhao S. Hu Y. Guo A. (2021). Effects of habitat types on the dynamic changes in allocation in carbon and nitrogen storage of vegetation-soil system in sandy grasslands: how habitat types affect C and N allocation? *Ecol. Evol.* 11 9079–9091. 10.1002/ece3.7751 34257945 PMC8258200

[B41] Mao W. Allington G. Li Y.-L. Zhang T.-H. Zhao X.-Y. Wang S.-K. (2012). Life history strategy influences biomass allocation in response to limiting nutrients and water in an arid system. *Polish J. Ecol.* 60 545–557.

[B42] Prober S. M. Leff J. W. Bates S. T. Borer E. T. Firn J. Harpole W. S. (2015). Plant diversity predicts beta but not alpha diversity of soil microbes across grasslands worldwide. *Ecol. Lett.* 18 85–95. 10.1111/ele.12381 25430889

[B43] Ramirez K. S. Craine J. M. Fierer N. (2012). Consistent effects of nitrogen amendments on soil microbial communities and processes across biomes. *Glob. Change Biol.* 18 1918–1927. 10.1111/j.1365-2486.2012.02639.x

[B44] Sayer E. J. Heard M. S. Grant H. K. Marthews T. R. Tanner E. V. J. (2011). Soil carbon release enhanced by increased tropical forest litterfall. *Nat. Clim. Change* 1 304–307. 10.1038/nclimate1190

[B45] Schiedung H. Tilly N. Huett C. Welp G. Brueggemann N. Amelung W. (2017). Spatial controls of topsoil and subsoil organic carbon turnover under C-3-C-4 vegetation change. *Geoderma* 303 44–51. 10.1016/j.geoderma.2017.05.006

[B46] Shen Y. Chen W. Q. Yang G. W. Yang X. Liu N. Sun X. (2016). Can litter addition mediate plant productivity responses to increased precipitation and nitrogen deposition in a typical steppe? *Ecol. Res.* 31 579–587. 10.1007/s11284-016-1368-5

[B47] Sokol N. W. Kuebbing S. E. Karlsen-Ayala E. Bradford M. A. (2019). Evidence for the primacy of living root inputs, not root or shoot litter, in forming soil organic carbon. *New Phytol.* 221 233–246. 10.1111/nph.15361 30067293

[B48] van Diepen L. T. A. Frey S. D. Landis E. A. Morrison E. W. Pringle A. (2017). Fungi exposed to chronic nitrogen enrichment are less able to decay leaf litter. *Ecology* 98 5–11. 10.1002/ecy.1635 28052385

[B49] Vasar M. Andreson R. Davison J. Jairus T. Moora M. Remm M. (2017). Increased sequencing depth does not increase captured diversity of arbuscular mycorrhizal fungi. *Mycorrhiza* 27 761–773. 10.1007/s00572-017-0791-y 28730541

[B50] Wang J. Zhao M. L. Willms W. Han G. D. Gao X. L. Wu Y. S. (2013). Productivity responses of different functional groups to litter removal in typical grassland of Inner Mongolia. *Acta Prataculturae Sinica* 22 31–38.

[B51] Wang J. Q. Shi X. Z. Zheng C. Y. Suter H. Huang Z. Q. (2021). Different responses of soil bacterial and fungal communities to nitrogen deposition in a subtropical forest. *Sci. Total Environ.* 755:142449. 10.1016/j.scitotenv.2020.142449 33045514

[B52] Wang L. Y. Zhou G. N. Zhu X. Y. Gao B. J. Xu H. D. (2021). Effects of litter on soil organic carbon and microbial functional diversity. *Acta Ecologica Sinica* 41 2709–2718. 10.5846/stxb202005141233

[B53] Wang S. K. Zuo X. A. Awada T. Medima-Roldán E. Feng K. T. Yue P. (2021). Changes of soil bacterial and fungal community structure along a natural aridity gradient in desert grassland ecosystems. *Inner Mongolia. Catena* 205:105470. 10.1016/j.catena.2021.105470

[B54] Wang S. K. Zuo X. A. Zhao X. Y. Awada T. Luo Y. Q. Li Y. Q. (2018). Dominant plant species shape soil bacterial community in semiarid sandy land of northern China. *Ecol. Evol.* 8 1693–1704. 10.1002/ece3.3746 29435244 PMC5792618

[B55] Wang X. Xu Z. W. Lu X. T. Wang R. Z. Cai J. P. Yang S. (2017). Responses of litter decomposition and nutrient release rate to water and nitrogen addition differed among three plant species dominated in a semi-arid grassland. *Plant Soil* 418 241–253. 10.1007/s11104-017-3288-8

[B56] Wardle D. A. Bardgett R. D. Klironomos J. N. Setala H. van der Putten W. H. Wall D. H. (2004). Ecological linkages between aboveground and belowground biota. *Science* 304 1629–1633. 10.1126/science.1094875 15192218

[B57] Yang F. Wu J. J. Zhang D. D. Chen Q. Zhang Q. Cheng X. L. (2018). Soil bacterial community composition and diversity in relation to edaphic properties and plant traits in grasslands of southern China. *Appl. Soil Ecol.* 128 43–53. 10.1016/j.apsoil.2018.04.001

[B58] Yang L. L. Gong J. R. Liu M. Yang B. Zhang Z. H. Luo Q. P. (2017). Advances in the effect of nitrogen deposition on grassland litter decomposition. *Chin. J. Plant Ecol.* 41 894–913. 10.17521/cjpe.2017.0023

[B59] Yue K. Peng Y. Peng C. H. Yang W. Q. Peng X. Wu F. Z. (2016). Stimulation of terrestrial ecosystem carbon storage by nitrogen addition: a meta-analysis. *Sci. Rep.* 6:19895. 10.1038/srep19895 26813078 PMC4728605

[B60] Zhan J. Li Y. L. Han D. Yang H. L. (2019). Effect of grazing on vegetation community and soil of lowland in the Hunshandake sandy land. *J. Desert Res.* 39 184–191.

[B61] Zhang A. (2019). Effects of litter addition and removal on the recruitment of seedlings in a semiarid grassland in northern China. *Chin. J. Appl. Environ. Biol.* 25 1286–1291.

[B62] Zhang T. A. Luo Y. Q. Chen H. Y. H. Ruan H. H. (2018). Responses of litter decomposition and nutrient release to N addition: a meta-analysis of terrestrial ecosystems. *Appl. Soil Ecol.* 128 35–42. 10.1016/j.apsoil.2018.04.004

[B63] Zhao H. L. Li J. Liu R. T. Zhou R. L. Qu H. Pan C. C. (2014). Effects of desertification on temporal and spatial distribution of soil macro-arthropods in Horqin sandy grassland, Inner Mongolia. *Geoderma* 223 62–67. 10.1016/j.geoderma.2014.01.026

[B64] Zhao H. L. Toshiya O. Li Y. L. Zuo X. A. Huang G. Zhou R. L. (2008). Effects of human activities and climate changes on plant diversity in Horqin sandy grassland, Inner Mongolia. *Acta Prataculturae Sinica* 17 1–8.

[B65] Zhao X. Li Y. Xie Z. Li P. (2020). Effects of nitrogen deposition and plant litter alteration on soil respiration in a semiarid grassland. *Sci. Total Environ.* 740:139634. 10.1016/j.scitotenv.2020.139634 32559551

[B66] Zhao X. X. Li Y. L. Li Y. W. Ju T. Z. (2020). Effects of increased nitrogen deposition and anthropogenic perturbation on soil respiration in a semiarid grassland. *Trans. Chin. Soc. Agric. Eng.* 36 120–127.

[B67] Zhou J. Gao Y. Wang Y. Zhao Y. J. (2021). The effect of different afforestation tree species on plant diversity after 50 years on mount TAI, CHINA. *Appl. Ecol. Environ. Res.* 19 4515–4526. 10.15666/aeer/1906_45154526

[B68] Zhou Z. H. Wang C. K. Zheng M. H. Jiang L. F. Luo Y. Q. (2017). Patterns and mechanisms of responses by soil microbial communities to nitrogen addition. *Soil Biol. Biochem.* 115 433–441. 10.1016/j.soilbio.2017.09.015

[B69] Zong N. Zhao G. S. Shi P. L. (2019). Different sensitivity and threshold in response to nitrogen addition in four alpine grasslands along a precipitation transect on the Northern Tibetan Plateau. *Ecol. Evol.* 9 9782–9793. 10.1002/ece3.5514 31534693 PMC6745826

[B70] Zuo X. A. Wang S. K. Lv P. Zhou X. Zhao X. Y. Zhang T. H. (2016). Plant functional diversity enhances associations of soil fungal diversity with vegetation and soil in the restoration of semiarid sandy grassland. *Ecol. Evol.* 6 318–328. 10.1002/ece3.1875 26811795 PMC4716495

[B71] Zuo X. A. Zhang J. Lv P. Wang S. K. Yang Y. Yue X. Y. (2018). Effects of plant functional diversity induced by grazing and soil properties on above and belowground biomass in a semiarid grassland. *Ecol. Ind.* 93 555–561. 10.1016/j.ecolind.2018.05.032

[B72] Zuo X. A. Zhao X. Y. Wang S. K. Li Y. Q. Lian J. Zhou X. (2012). Influence of dune stabilization on relationship between plant diversity and productivity in Horqin Sand Land, Northern China. *Environ. Earth Sci.* 67 1547–1556. 10.1007/s12665-012-1950-2

