# Supplementary material for: Effects of nitrogen addition and plant litter manipulation on soil fungal and bacterial communities in a semiarid sandy land
Source: Front Microbiol. 2023 Mar 27;14:1013570. doi: 10.3389/fmicb.2023.1013570 (PMC10083410; doi:10.3389/fmicb.2023.1013570)
Supplement: Supplementary file 2 [file Data_Sheet_1.docx]

Supplementary Figure 1. Plot of sample plot


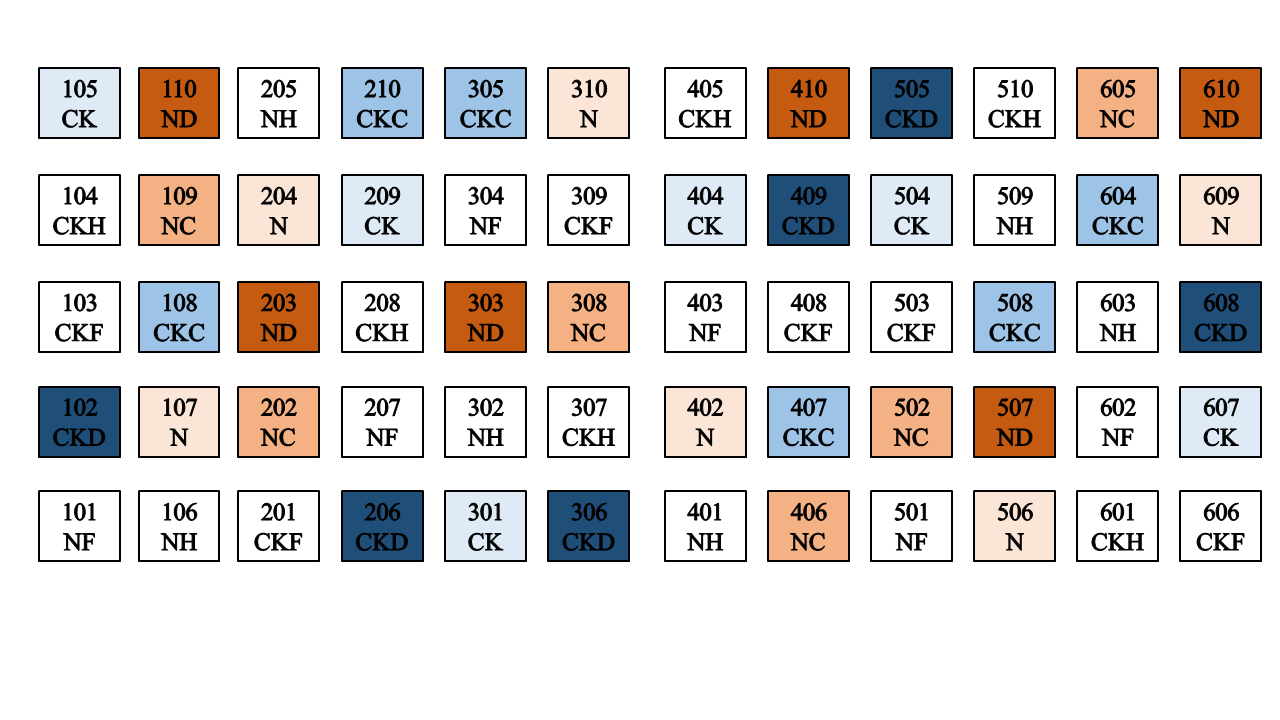


Note: CK, mean control; CKC, mean mowing; CKD, mean litter doubling; CKF, mean burning; CKH, mean enclosure; N, mean nitrogen addition; NC, mean nitrogen addition and mowing; ND, mean nitrogen addition and litter doubling; NF, mean nitrogen addition; NH, mean nitrogen addition and enclosure;

Supplementary Figure 2. The 6-year of plant community diversity indexes in N addition and litter manipulation treatments during 2014–2019. Significance levels were presented to show the effect of altered litter input (C) and N addition (N) treatments and their interaction (C × N) on these parameters (ns, *p* > 0. 1; #, *p* < 0.1; *, *p* < 0.05; **, *p* < 0.01; ***, *p* < 0.001). The letters in the bar graph indicate the results of multiple comparisons among different litter input and nitrogen addition treatments. Different lowercase letters indicate significant difference among three altered litter input treatments (*p* < 0.05), and uppercase letters indicate the significant difference between two nitrogen treatments (*p* < 0.05).

Supplementary Figure 3. The 6-year of soil fungal and bacterial simpson diversity index and ACE richness in N addition and litter manipulation treatments during 2014–2019. Significance levels were presented to show the effect of N addition (N) and altered litter input (C) treatments and their interaction (N × C) on these parameters (ns, *p* > 0. 1; #, *p* < 0.1; *, *p* < 0.05; **, *p* < 0.01; ***, *p* < 0.001). The letters in the bar graph indicate the results of multiple comparisons among different litter input and nitrogen addition treatments. Different uppercase letters indicate the significant difference between two nitrogen treatments (*p* < 0.05).

Supplementary Table 1. Analysis of similarities (ANOSIM) of soil fungal and bacterial community under N addition and litter manipulation during 2014–2019.

| **Different groups** | **Fungi** | | | **Bacteria** | | | |  |
| --- | --- | --- | --- | --- | --- | --- | --- | --- |
|  | **R** | **P_value** | **Sig** | | **R** | **P_value** | **Sig** | |
| N0C1/N0C0 | 0.131 | 0.027 | * | | -0.1463 | 0.937 |  | |
| N0C1/N0C2 | 0.087 | 0.125 |  | | 0.020 | 0.365 |  | |
| N0C1/N10C1 | 0.356 | 0.006 | ** | | 0.285 | 0.01 | * | |
| N0C1/N10C0 | 0.670 | 0.002 | ** | | 0.539 | 0.005 | ** | |
| N0C1/N10C2 | 0.360 | 0.005 | ** | | 0.402 | 0.006 | ** | |
| N0C0/N10C2 | 0.213 | 0.029 | * | | 0.165 | 0.106 |  | |
| N0C0/N10C1 | 0.474 | 0.004 | ** | | 0.339 | 0.001 | ** | |
| N0C0/N10C0 | 0.785 | 0.003 | ** | | 0.650 | 0.003 | ** | |
| N0C0/N10C2 | 0.675 | 0.002 | ** | | 0.550 | 0.003 | ** | |
| N0C2/N10C1 | 0.507 | 0.003 | ** | | 0.289 | 0.006 | ** | |
| N0C2/N10C0 | 1.000 | 0.003 | ** | | 0.605 | 0.002 | ** | |
| N0C2/N10C2 | 0.805 | 0.004 | ** | | 0.372 | 0.011 | * | |
| N10C1/N10C0 | 0.026 | 0.278 |  | | -0.024 | 0.589 |  | |
| N10C1/N10C2 | -0.152 | 0.914 |  | | -0.011 | 0.479 |  | |
| N10C0/N10C2 | 0.400 | 0.011 | * | | -0.030 | 0.554 |  | |
| N0/N10 | 0.554 | 0.001 | ** | | 0.412 | 0.001 | ** | |

*, *p* < 0.05; **, *p* < 0.01; ***, *p* < 0.001.

Supplementary Table 2. Results of multi-factor variance analysis of the effects of N addition and litter manipulation on soil microbial community proportion at phyla level

| **kingdom** | **Phyla level** | **N** | | **C** | | **N*C** | |
| --- | --- | --- | --- | --- | --- | --- | --- |
|  |  | F | P | F | P | F | P |
| **Fungi** | Ascomycota | 5.046 | 0.032 | 1.331 | 0.279 | 0.163 | 0.850 |
|  | Basidiomycota | 2.384 | 0.133 | 2.326 | 0.115 | 0.772 | 0.471 |
|  | Mortierellomycota | 2.573 | 0.119 | 2.081 | 0.142 | 0.088 | 0.916 |
|  | Chytridiomycota | 12.923 | 0.001 | 3.294 | 0.051 | 3.102 | 0.060 |
|  | Kickxellomycota | 2.237 | 0.145 | 2.058 | 0.145 | 1.860 | 0.173 |
|  | Glomeromycota | 0.000 | 0.987 | 3.946 | 0.030 | 3.731 | 0.036 |
|  | Mucoromycota | 1.611 | 0.214 | 2.265 | 0.121 | 0.342 | 0.713 |
|  | Rozellomycota | 4.059 | 0.053 | 5.583 | 0.009 | 5.212 | 0.011 |
|  | Zoopagomycota | 1.802 | 0.190 | 1.121 | 0.339 | 1.243 | 0.303 |
|  | Blastocladiomycota | 0.569 | 0.456 | 0.762 | 0.476 | 1.692 | 0.201 |
| **Bacteria** | Proteobacteria | 5.938 | 0.021 | 1.329 | 0.280 | 1.079 | 0.353 |
|  | Actinobacteria | 1.245 | 0.273 | 0.580 | 0.566 | 1.072 | 0.355 |
|  | Acidobacteria | 21.190 | 0.000 | 1.370 | 0.270 | 0.650 | 0.529 |
|  | Firmicutes | 6.984 | 0.013 | 1.177 | 0.322 | 0.981 | 0.387 |
|  | Bacteroidetes | 6.286 | 0.018 | 1.593 | 0.220 | 1.582 | 0.222 |
|  | Gemmatimonadetes | 0.070 | 0.793 | 0.241 | 0.787 | 0.079 | 0.924 |
|  | Thaumarchaeota | 5.190 | 0.030 | 0.014 | 0.986 | 0.511 | 0.605 |
|  | Chloroflexi | 0.098 | 0.756 | 0.405 | 0.671 | 0.806 | 0.456 |
|  | Verrucomicrobia | 3.917 | 0.057 | 0.727 | 0.492 | 1.324 | 0.281 |
|  | Spirochaetes | 0.752 | 0.393 | 0.842 | 0.441 | 1.083 | 0.351 |

Supplementary Figure 4. The 6-year of soil fungal community proportion at phyla level in N addition and litter manipulation treatments during 2014–2019. Significance levels were presented to show the effect of altered litter input (C) and N addition (N) treatments and their interaction (C × N) on these parameters (ns, *p* > 0. 1; #, *p* < 0.1; *, *p* < 0.05; **, *p* < 0.01; ***, *p* < 0.001). The letters in the bar graph indicate the results of multiple comparisons among different litter input and nitrogen addition treatments. Different uppercase letters indicate the significant difference (*p* < 0.05).

Supplementary Figure 5. The 6-year of soil bacterial community proportion at phyla level in N addition and litter manipulation treatments during 2014–2019. Significance levels were presented to show the effect of altered litter input (C) and N addition (N) treatments and their interaction (C × N) on these parameters (ns, *p* > 0. 1; #, *p* < 0.1; *, *p* < 0.05; **, *p* < 0.01; ***, *p* < 0.001). The letters in the bar graph indicate the results of multiple comparisons among different litter input and nitrogen addition treatments. Different uppercase letters indicate the significant difference (*p* < 0.05).

Supplementary Figure 6. The correlation analysis of plant community diversity with soil microbial alpha diversity and community composition in N addition and litter manipulation treatments during 2014–2019. The correlation was evaluated by Pearson correlation coefficient. Blue indicates a negative correlation, and red indicates a positive correlation, and the strength of color reflects the strength of the correlation.
